# Varietal Resistance and Chemical Ecology of the Rice Stink Bug, *Oebalus pugnax*, on Rice, *Oryza sativa*

**DOI:** 10.3390/plants11223169

**Published:** 2022-11-19

**Authors:** Santhi Bhavanam, Michael J. Stout

**Affiliations:** Department of Entomology, Louisiana State University Agricultural Center, Baton Rouge, LA 70803, USA

**Keywords:** host-plant resistance, silicon, metathoracic glands, dorsal abdominal glands

## Abstract

The rice stink bug, *Oebalus pugnax* F. (Hemiptera: Pentatomidae), is a key pest of heading rice in the southern United States. Chemical insecticide application is currently the primary method of control of *O. pugnax*, warranting an improved management program for this species. The potential other management tactics for *O. pugnax* include eco-friendly measures such as host-plant resistance, silicon application, and the use of semiochemicals. In this study, the feeding preference and performance of *O. puganx* on cultivated and non-cultivated rice varieties were examined. Choice tests showed that the rice varieties Cheniere and Kaybonnet were most and least preferred by *O. pugnax* for feeding, respectively. The results of a no-choice experiment showed that the number of nymphs surviving to the adult stage did not differ among rice varieties, although the percent survival was low on the varieties Kaybonnet and Jazzman. Here, we also showed for the first time that silicon application had a significant negative impact on *O. pugnax* performance, increasing the nymph development time and reducing survival by almost 40% relative to the control. Based on these results, it could be suggested that silicon amendment is a promising management strategy for this pest. Further research is needed to examine whether silicon application also reduces the feeding damage caused by *O. puganx*. In addition, the chemical compositions of the metathoracic gland and dorsal abdominal gland extracts were also characterized for the first time in this study, and their biological roles and potential use in pest management are discussed.

## 1. Introduction

Rice, *Oryza sativa* L. (Poales: Poaceae), is an important staple crop that is cultivated in different regions of the world, including the United States [1]. Among rice insect pests in the United States, the rice stink bug, *Oebalus pugnax* F. (Hemiptera: Pentatomidae), is important as a pest of heading rice [2]. Adults and nymphs feed on developing rice grains from anthesis to the end of the hard dough stage [3,4]. Feeding by *O. pugnax* results in reductions in rough and head rice yields and also in grain quality [3,5]. Chemical insecticides (pyrethroids and neonicotinoids) are effective at controlling *O. pugnax.* However, sole reliance on insecticides may be leading to the development of resistance in some populations of *O. pugnax* [6,7]; in addition, insecticide use has negative impacts on natural enemies and other non-target organisms [8,9]. All these issues necessitate the investigation of other eco-friendly control tactics to develop a sustainable management program for *O. pugnax.*

Host-plant resistance is an important pest management tool that is usually compatible with other control measures, is environmentally safe, and often reduces insecticide use because resistant varieties tend to harbor lower pest populations and/or help keep insect damage below economic thresholds [10]. Plant resistance can affect insect behavior, resulting in the reduced acceptance and colonization of the host plant, which is referred to as antixenosis [10]. Antibiosis, in contrast, is a type of plant resistance that impacts insect biology and physiology, resulting in impaired development, survival, or reproduction [10]. Plant resistance studies conducted in the past found evidence of antibiosis in some rice genotypes against *O. puganx* [11] and differences in damage levels and subsequent yield losses caused by *O. pugnax* among rice varieties [12]. However, no studies have evaluated the preference and performance of *O. pugnax* on currently cultivated rice varieties.

Silicon application to plants is an economical and eco-friendly alternative to insecticides that has been proven to increase the resistance of plants against herbivores through cell silicification and formation of physical barriers (biomechanical defenses) or by eliciting plant defense pathways (biochemical defenses) [13,14]. Rice actively absorbs silicon, which is deposited in various plant parts including the culms, leaves, and hulls, and silicon constitutes about 10% of the rice total dry weight [15]. In rice, silicon amendment confers resistance against leaf feeders, sap-sucking insects, and stem borers [16,17,18]. Rice hulls contain 1% silicon by dry weight and silicon supplementation increases the silicon content in hulls to 8% [19,20]. However, to our knowledge, no study has examined the effects of silicon application on hemipteran bugs that infest rice grains, in particular *O. pugnax*.

Adult *O. pugnax*, unlike nymphs, are capable of flight and frequently migrate into heading rice fields, switching between host plants at appropriate phenological stages for feeding [2,21]. Adults are also prone to flight following insecticide application. Semiochemicals that can influence insect behavior can be used as attractants or repellants in the form of trap baits or trap crops to manage dispersing pest populations [22]. Additionally, semiochemicals derived from plants or insects are generally not harmful to the environment or other non-target organisms. The use of semiochemicals has been suggested as a potential alternative management measure for stink bugs.

Most hemipterans, including *O. pugnax*, release a pungent-smelling secretion when under stress; these secretions are produced in the metathoracic glands (MTGs) in adults and the dorsal abdominal glands (DAGs) in nymphs [23,24,25]. The contents of the MTGs and DAGs of other hemipterans are mixtures of aldehydes, esters, ketones, alkenes, alkanes, and oxo-2-hexenals [24,26,27]. The MTG contents differ quantitatively and qualitatively between males and females [28,29] and from the DAG extracts of nymphs, which also differ among instars [26,27]. The contents of the MTG and DAG are involved in intra- and inter-specific communication. Research has just begun to assess the application of MTG and DAG extracts/compounds as pest management tools in other stink bugs [29,30,31,32]. However, little is known about the contents of the DAGs and MTGs of *O. pugnax*. Therefore, the characterization of the compounds of these glands is the first step in conducting future research and developing a semiochemical-based management program for *O. pugnax*.

Bhavanam, et al. [33] identified several areas in need of further investigation for the development of improved management strategies for *O. pugnax*. Using a combination of pest control strategies that target different life stages would be a promising approach for the management of *O. pugnax*. Host-plant resistance has been an important management tool for various key pests of rice. Silicon-mediated plant resistance has been effective in controlling pests and diseases of agricultural crops. Semiochemicals are also used for pest management. The use of semiochemicals is usually moderately effective on its own, and is often integrated with other control measures for better pest control [34,35]. In order to use these potential management tactics against *O. pugnax*, a better understanding of the feeding preference and biology of this species on different rice varieties and the chemical ecology of the species is required. Therefore, this study was initiated with the aim to investigate (1) feeding preference of *O. pugnax* for different rice varieties using choice tests; (2) the growth and development of *O. pugnax* on eight different rice varieties using no-choice tests; (3) the effects of silicon soil amendment on the performance of *O. pugnax*; and (4) the chemical compounds of the DAG extracts of all instars and MTG secretions of adults.

## 2. Results

### 2.1. Varietal Preference Study

**Figure 1:**
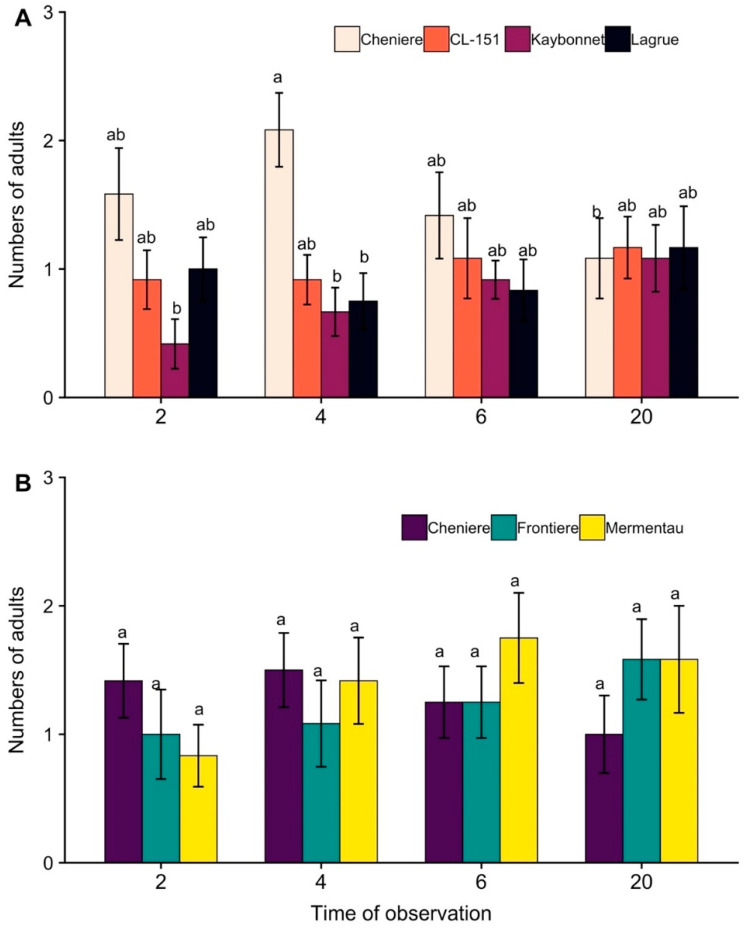
Mean (±SE) number of adult rice stink bugs, *Oebalus pugnax*, observed on different varieties of rice at 2, 4, 6, and 20 h post-adult release in (**A**) first group and (**B**) second group of varieties. In each figure, bars with different lowercase letters indicate significant differences among means (*p* < 0.05).

The results of the choice assay with the first group of varieties showed a significant interaction between variety and time (F_9, 132_ = 2.79, *p* = 0.005). There was a marginal difference in the mean number of adults seen on different rice varieties (F_3, 44_ = 2.60, *p* = 0.064). The mean number of adults observed on Cheniere at 4 h post-adult release (hpr) was significantly higher compared to the numbers observed on Kaybonnet and LaGrue at 4 hpr and Kaybonnet at 2 hpr (Figure 1A). Similarly, for the choice assay with the second group of varieties, the interaction between variety and time was significant (F_6, 99_ = 2.70, *p* = 0.018), but a post hoc analysis yielded no significant differences among the means (Figure 1B). Neither variety (F_2, 33_ = 0.10, *p* = 0.907) nor time (F_3, 99_ = 1.83, *p* = 0.147) had a significant effect on the preference of *O. pugnax*.

### 2.2. Varietal Performance Study

The *O. pugnax* nymph development time (F_7,35_ = 0.68, *p* = 0.692; Table 1), the percent survival to adult stage (F_7,35_ = 0.82, *p* = 0.578; Table 1), and the fresh weights of adult males (F_7,34_ = 1.53, *p* = 0.189; Table 1) and females (F_7,34_ = 1.34, *p* = 0.264; Table 1) did not differ significantly among the different varieties tested.

### 2.3. Silicon Study

**Figure 2:**
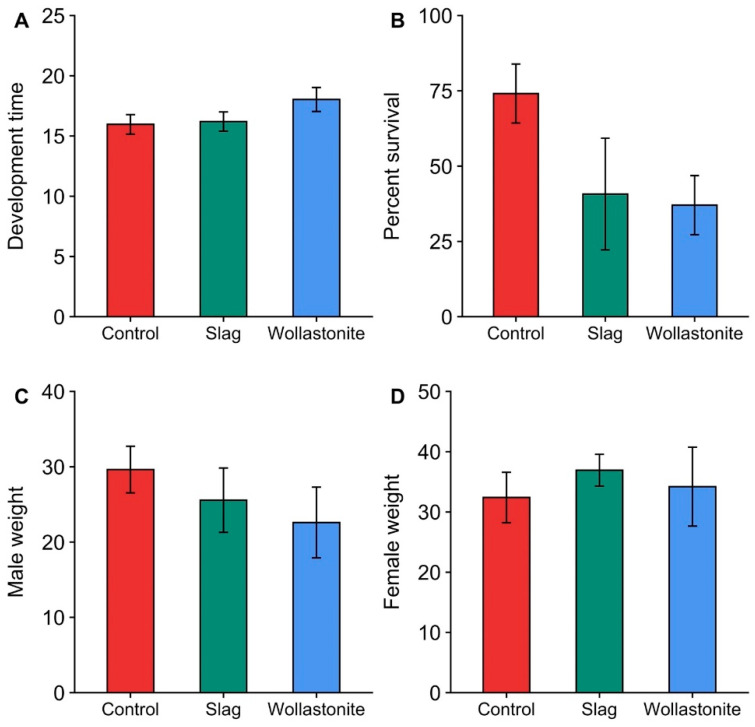
Means (±SE) of (**A**) nymph development time (days), (**B**) survival to adult stage (%), and adult weights (mg) of 1 d-old (**C**) male and (**D**) female *Oebalus pugnax* reared on panicles taken from rice (*Oryza sativa*) plants treated with silicon (@ 1120 kg/ha) provided in the form of slag, wollastonite, or untreated control in the laboratory at 27 ± 1 °C and 14:10 h L:D photoperiod.

There was a significant effect of silicon treatment on the *O. pugnax* nymph development time (F_2,4_ = 7.37, *p* = 0.046; Figure 2A). Nymphs fed on panicles taken from wollastonite-treated plants took, on average, 2 days longer to emerge as adults compared to nymphs reared on panicles taken from untreated plants (*p* = 0.05). Silicon soil amendment to rice plants had no significant effect on the number of nymphs surviving to the adult stage (F_2,4_ = 3.65, *p* = 0.126; Figure 2B) or the adult fresh weights of males (F_2,3_ = 5.71, *p* = 0.095; Figure 2C) and females (F_2,4_ = 0.73, *p* = 0.536; Figure 2D).

### 2.4. Chemical Ecology of Dorsal Abdominal Gland and Metathoracic Gland Extracts

The DAG extracts of *O. pugnax* nymphs contained a mixture of alkanes, aldehydes, oxo-alkenals, and monoterpenoids (Table 2). Four compounds, dodecane, tridecane, tetradecanal, and (*E*)-4-oxo-2-hexenal, were detected in the DAG extracts of all instars, although their relative abundances differed among instars (Table 2). In the DAG extracts of the first instars, (*E*)-4-oxo-2-hexenal was the most abundant compound, followed by tridecane and 2-undecenal, both of which were present in equal proportions. In the DAG extracts of the second through fifth instars, the first-, second-, and third-most abundant compounds were (*E*)-4-oxo-2-hexenal, tridecane, and tetradecenal, respectively. These three compounds combined accounted for more than 90% of the total DAG extracts. Additionally, the DAG extracts of different instars differed qualitatively: (*E*)-2-hexenal was present in the DAG extracts of the third to fifth instars, but not in the other instars, and minor proportions of linalool and α-terpineol were found in the DAG extracts of first instars (Table 2).

The compounds identified in the MTG extracts of males and females are presented in Table 3. The contents of the MTG were qualitatively similar for both sexes except for a few differences in the compounds present in minor proportions (Table 3). However, the relative abundance of the compounds in the MTG extracts differed substantially between males and females. Tridecane was the most abundant compound in both sexes.

## 3. Discussion

### 3.1. Varietal Preference Study

The first aim of this study was to determine the feeding preference of *O. pugnax* for different rice varieties. The results of the choice tests showed significant differences in feeding preference among rice varieties. The variety Cheniere, which is widely cultivated in the southern United States, was the most preferred and the variety Kaybonnet, an older variety that is no longer widely grown, was the least preferred (Figure 1A). The feeding preference did not vary among other varieties that are currently cultivated, but a pattern in preference was observed (Figure 1). Cheniere harbored more insects at 2 and 4 hpr, but the number of insects on this variety decreased thereafter in both groups. The number of adults found on Frontiere and Mermentau gradually increased with time, and more insects on these two varieties were detected at 6 and 20 hpr (Figure 1B). The mean number of insects found on CL151 was constant at all time points (Figure 1A).

While choosing a host plant, insects initially rely on visual and olfactory cues [36]. Singh et al. [37] showed that Kaybonnet emits relatively high amounts of methyl salicylate (Me-SA) and limonene; both compounds are known to deter feeding and repel insects [38,39,40]. Corroborating this, we observed fewer insects on Kaybonnet, especially at 2 and 4 hpr. Here, the panicles were used in choice tests within 20 min of their excision. It is likely that the excised panicles may have contained and released large amounts of volatiles, especially at the start of the choice test, which may have contributed to the non-preference for this variety. Additional research on the volatiles emitted by panicles at various stages of development are needed.

After contact with the host, chemical and tactile cues mediated by plant morphology influence insect behavior and play an important role in the antixenosis of host plants [36]. In rice, varietal susceptibility or resistance to insects that feed on grains in the field and in storage is often mediated by the grain’s morphological/physiological traits, such as the cell wall composition, thickness, gap between palea and lemma, hull integrity, length-to-width ratio of the kernel, and plant volatiles [41,42,43,44,45]. Host plant acceptance is also influenced by the presence of feeding deterrents and stimulants [36,46]. For example, nutrients such as starch and amylopectin in rice grains, depending on their concentration, can stimulate feeding in mirid bugs, *Trigonotylus caelestialium* Kirkaldy and *Stenotus rubrovittattus* Matsumura [47], and rice weevils, *Sitophilus oryzae* L. [48]. Grain morphological characteristics, physiological characteristics, and nutrient quantity and quality often differ among rice varieties [49,50,51], and further research needs to be conducted to identify the factors that influence the feeding preference of *O. pugnax*; this knowledge may be used to develop attractants or varieties that are less susceptible to *O. pugnax* feeding.

### 3.2. Varietal Performance Study

The survival of *O. pugnax* nymphs fed on different rice varieties ranged between 75 and 92%. Although the differences in performance on different varieties were not significant, the percent survival was lowest on Kaybonnet, followed by Jazzman (Table 1), and most of the mortality occurred during the second instar on these two varieties (personal observation). A higher mortality of early instars has also been reported in other stink bugs that infest soybeans, and this effect has been linked to the morphological and physiological characteristics of pods that prevent the stylet of young nymphs from reaching the seed to draw nutrients [52,53]. In rice, composition, thickness, and hardiness of hulls can affect insect stylet penetration and limit feeding, resulting in lower insect fitness [41,43,45]. Moreover, plant volatiles often have a detrimental effect on insect performance. As noted above, Kaybonnet emits Me-SA and limonene, while Jazzman, an aromatic variety, contains a volatile profile that is qualitatively and quantitively different from that of other regular rice varieties.

Insects fed on foods high in protein tend to grow faster and become larger [54], but not always [55,56]. Here, the nymphs of *O. pugnax* that were fed on Frontiere, a high-protein rice variety that contains an average of 11% protein at maturity [49], had development times, survival rates, and adult weights that were comparable to those of the nymphs fed on Cheniere and CL151. The lack of increased performance of *O. pugnax* on Frontiere may be related to the timing of protein synthesis. Protein formation usually starts 5 days after anthesis, progresses with age, reaches a peak at 12 days from anthesis, and then plateaus [57]. This study used 7–12-day-old panicles, and it is possible that the protein bodies may have been in their initial stages of development and hence there was no impact on *O. pugnax* performance. Moreover, protein bodies, which are usually embedded between starch granules [57], may not be easily accessible to *O. pugnax* nymphs. Alternatively, the seeds of all varieties may have contained sufficiently high concentrations of nutrients. Furthermore, insects are capable of regulating their protein and carbohydrate intake, and when given a choice, they consume an optimal amount of nutrients required for their growth and development [58,59]. Therefore, a relatively high protein content in Frontiere may not have any positive impact on *O. pugnax* performance.

### 3.3. Silicon Study

This is the first study to demonstrate that silicon application has a negative impact on *O. pugnax* performance. The nymphs fed on panicles from wollastonite-treated plants took, on average, two additional days to emerge as adults compared to the nymphs that were fed on panicles from control plants (Figure 2A). Additionally, this study also found a non-significant reduction in the number of nymphs that survived to the adult stage by approximately 40% due to the silicon treatment relative to control (Figure 2B). To our knowledge, the research conducted by de Souza et al. [60] is the only other study that has investigated the effects of silicon amendment on stink bugs that feed on reproductive parts. These authors observed similar results: silicon application prolonged the nymph development time of the neotropical brown stink bug, *Euschistus heros* F., in soybean, *Glycine max* F.

The reduced performance of *O. pugnax* nymphs on silicon-treated plants indicates that the nymphs did not have access to a sufficient supply of the nutrients required for their normal growth and development. This could be because (1) silicon deposited in the hulls formed a mechanical barrier that restricted or reduced the penetration of stylets to draw the nutrients from grains, or (2) silicon increased the activities of plant defense enzymes that are involved in the biosynthesis of secondary metabolites that have antiherbivore and antinutritive properties [14,61]. For example, lignin in the cell wall of rice grains formed a physical barrier that limited insect feeding and thus conferred resistance against rice ear bugs, *Leptocorisa chinensis* Dallas and *Cletus punctiger* Dallas [41,43].

From a pest management perspective, a 40% reduction in nymph survival due to the silicon application may translate into less feeding activity, which may result in less damage and in turn lower yield losses and peck incidence due to *O. pugnax*. Past research has shown that the feeding rates of nymphs were comparable to those of adults [62]. Nymph feeding results in the formation of peck, and the degree of damage caused by nymph feeding is positively related to nymph infestation levels [63,64]. Moreover, the rate of grain development is dependent on temperature; under field conditions, long-grain varieties generally take 21 days from anthesis to reach the hard dough stage, and within 35 days, all panicles of a single plant mature [65]. Silicon application prolonged nymph development by 2 days, and as a result, the total development time, when combined with the egg stage (4 days) and the first instar stage (2 days), was 23 days on silicon-treated plants compared to 21 days on control plants. Because of this increased development time, the development of the later instars may coincide with the hard dough stage, which is a stage that is less suitable and thus less preferred for feeding by *O. pugnax* [64]. Considering the fact that nymphs cannot fly and thus cannot easily move to a different plant, delays in development may have a direct negative impact on *O. pugnax* survival or adult body mass and, in turn, population densities, and may indirectly affect the incidence of peck. Even though the fourth and fifth instars cause peck, by the time nymphs reach this stage, the panicles would be in the later stages of hard dough, which is the stage when susceptibility to peck incidence is lower. Cato et al. [66] have shown that, for panicles with 20, 30, and 60% of grains at the hard dough stage, formation of peck due to *O. pugnax* feeding was lowest on the latter while highest on the former. Further work should be undertaken to document the reductions in the damage caused by *O. pugnax* in plants treated with silicon in the field.

### 3.4. Chemical Ecology of Dorsal Abdominal Glands and Metathoracic Glands

Four compounds, (*E*)-4-oxo-2-hexenal, dodecane, tridecane, and tetradecanal, were detected in the DAG extracts of all instars of *O. pugnax.* In addition, GC–MS analysis also showed qualitative differences and variation in the relative abundances of the compounds among the DAG extracts of different instars (Table 2), as reported for the DAGs of nymphs of other hemipteran species [67,68]. The contents of the MTGs of *O. pugnax* males and females was a mixture of alkanes, alkenes, aldehydes, alcohols, and esters (Table 3), which is a composition typical of the MTG secretions of other pentatomids, coreids, and lygaeids [27]. The composition and function of MTG secretions have been investigated in other hemipteran species.

The most abundant compound in the DAG extracts of all instars, (*E*)-4-oxo-2-hexenal, has been reported as a defense compound against predators [69]. For example, (*E*)-4-oxo-2-hexenal deterred jumping ants, *Harpegnathos saltator* Jerdon [70], caused paralysis and death of crickets, *Acheta domesticus* L. [71], and was toxic to the California mantis *Stagmomantis californica* Rehn and Hebard [72]. Nilakhe [73] reported that the egg masses and nymphs of *O. pugnax* are predated by long- and short-horned grasshoppers in field situations. Therefore, the presence of this compound in the DAG extracts of *O. pugnax* may be advantageous, as nymphs are not capable of flight.

Tridecane present in the MTGs of adults and the DAG extracts of nymphs serves as an aggregation pheromone, an alarm pheromone, a solvent that aids in the penetration and retention of other MTG compounds on insect cuticles, and a synergist for aldehydes in MTG contents [74,75]. This study also shows that tridecane is relatively less abundant in the DAG extracts of first instars. In most pentatomids, including *O. pugnax*, first instars cluster around the egg shells after hatching until molting to the next stage to protect themselves from desiccation and natural enemies [76]. Lockwood and Story [77] showed that aggregating first instars of the southern green stink bug, *Nezara viridula* L., had higher survival rates and developed faster compared to non-aggregating individual nymphs at high temperatures and low humid conditions. In the same species, Lockwood and Story [78] also demonstrated that low doses of tridecane (5 × 10^−5^ µL) attracted first instars, resulting in their aggregation, while high doses (0.5 and 5 µL) of tridecane had a repellent effect and induced dispersal in first instars. Hamm [79] also reported a similar effect of tridecane on adult *O. pugnax*. Therefore, a possible explanation for the low levels of tridecane in first instars could be that the presence and release of large amounts of tridecane by *O. pugnax* first instars may interfere with their aggregation behavior and, in turn, their survival, or that high doses of tridecane may have a toxic effect that may lead to the death of the first instars. Gunawardena and Herath [80] investigated the role of tridecane as a fumigant and found that higher concentrations of tridecane had a toxic effect, causing mortality of the test species *Anoplolepis longipes* Smith, *Sitotroga cerealella* Olivier, and *Culex quinquefasciatus* Say.

The aldehydes present in the MTG secretions of adult hemipterans have diverse functions and serve as alarm pheromones, repellents, defenses, feeding stimulants, and aggregation pheromones [30,81,82,83,84]. Moreover, (*E*)-2-hexenal, (*E*)-2-octenal, (*E*)-2-decenal, tetradecanal, and D-limonene have fungistatic effects (inhibited conidia growth) and fungicidal effects (reduced spore germination) against the entomopathogenic fungi *Metarhizium anisopliaeon* and *Beauveria bassiana* [85,86,87], which may affect the efficacy of these entomopathogens. This study showed the DAG extracts of the third through fifth instars and adult MTG extracts contained relatively large amounts of aldehydes; however, the DAG extracts of first instars contained only minor amounts of tetradecanal and the monoterpenoids linalool and α-terpineol. Based on these results, it can be interpreted that early instars that contain smaller amounts of aldehydes may be susceptible to entomopathogenic fungi, and its use against early instars may provide desirable control of *O. pugnax*.

The role of (*E*)-2-hexenal, (*E*)-2-decenal, tridecane, and (*E*)-4-oxo-2-hexenal as kairomones has been established under lab conditions [32,75]. Vieira et al. [31] examined the attractiveness of (*E*)-2-hexenal to egg parasitoids in soybean fields and found that the installation of eggs treated with this aldehyde attracted the egg parasitoids *Telenomus* spp. and *Trissolcus* spp., which resulted in increased egg parasitization rates at one week and higher predation rates at seven weeks following installation. The egg parasitoid *Telenomus podisi* Ashmead is very efficient at controlling populations of *O. pugnax* [9,88]. Future research should focus on the identification and determination of the concentrations of the MTG compounds that are attractive to *T. podisi* and evaluate the influence of these compounds on *O. pugnax* egg parasitization rates by *T. podisi* under field situations.

## 4. Materials and Methods

### 4.1. Insect Rearing

The insects used in this study were taken from a colony of *O. pugnax* kept in an environmentally controlled room at 27 ± 1 °C, 14:10 h L:D photoperiod at Louisiana State University (LSU), Baton Rouge, LA. The colony was initially established with adults collected from rice plots at the H. Rouse Caffey Rice Research Station located in Crowley, LA. Adults were transported in mesh cages (30 × 30 × 30 cm) to the laboratory, where they were introduced into a glass aquarium (30 × 60 × 40 cm) lined with paper towels at the bottom and covered with a lid made of fine mesh for ventilation and to prevent insect escape. Adults were fed on rice panicles at R6 and R7 (milk and soft dough stages, respectively [89]) and barnyard grass seed heads. Panicles/seed heads were kept fresh by placing their cut ends in water in a conical flask. Food was changed every 3 days.

Egg masses deposited on rice panicles and wax paper placed along the lateral sides of the aquarium were collected and placed in a rearing tray (Frontier Agricultural Sciences, Newark, DE, USA) along with a moistened cotton ball and covered with a clear rearing tray lid (Frontier Agricultural Sciences, Newark, NJ, USA). First instars were not provided with food and remained in the same tray until molting to second instars. All other instars (2nd through 5th) were reared in transparent plastic boxes (17 × 8 × 4 cm) that contained a rice panicle at the R6 to R7 stages and closed with lid that had two holes covered with fine mesh. Only the portion of the rice panicle bearing the spikelets was placed inside the box; the remaining portion of the panicle was pushed outside through a hole on the lateral side of the box and the hole was sealed with a foam plug with a vertical slit. The cut end of the panicle was inserted into a floral tube containing water. Moistened germination paper was placed at the bottom of the box to maintain high humid conditions inside the box. Rice panicles and germination papers were replaced every 3 days. After emergence, adults were transferred to the aquarium.

### 4.2. Rice Varieties and Plants

The rice varieties tested in this study included one medium-grain variety, Jupiter, and seven long-grain varieties: Cheniere; CL151 (herbicide-tolerant variety); Frontiere (high-protein variety); Jazzman (an aromatic variety); Mermentau; and two long-grain varieties that are no longer cultivated, Kaybonnet and LaGrue. The two uncultivated long-grain varieties were chosen because they were reported to have some resistance to *O. pugnax* [12,37]. Kaybonnet and LaGrue were developed by the Arkansas Agricultural Experiment Station and USDA-ARS, and all the remaining varieties by LSUAgCenter. The selected rice varieties differed in their number of days to heading, and hence the rice planting dates were staggered to synchronize the heading dates of different varieties and thereby obtain panicles in the R6 and R7 stages at the same time. All varieties were planted weekly from May to July in a greenhouse.

Rice plants were grown in pots (Ø = 6”; height = 6”) containing a soil mix made of 2 parts top soil, 1 part peat, and 1 part sand. Five rice seeds were planted in each pot. At 10 days after planting, plants were thinned to one plant per pot and supplied with 1.5 g of 13:13:13 N:P:K controlled-release fertilizer (Carl Pool Products, Galveston, TX, USA). Plants were grown under ambient light and watered as needed. During the entire planting period, the day temperature in the greenhouse ranged between 27 and 32 °C. The rice panicles needed for the following studies were taken from these plants.

### 4.3. Varietal Preference Study

To determine the relative preference of *O. pugnax* for different rice varieties, choice tests were conducted under greenhouse conditions. The six rice varieties tested in this study were Cheniere, CL151, Frontiere, Kaybonnet, LaGrue, and Mermentau. These varieties were divided into two groups with Cheniere included in both groups. The first group consisted of Cheniere, CL151, Kaybonnet, and LaGrue, while the second group consisted of Cheniere, Frontiere, and Mermentau. Each group was tested separately and the procedure used to conduct the choice test was the same for both groups.

Choice tests were conducted in arenas constructed with a PVC cylinder (Ø = 60 cm; height = 30 cm) that contained four holes on the sides. The top of the cylinder was covered with a fine aluminum mesh that facilitated both insect observation and ventilation. On the day of the choice test, a single rice panicle at the R6/R7 stage was excised 10 cm below the axis node from a plant of each variety. Immediately, the cut end of the panicle was placed in a floral tube filled with water to reduce desiccation of the panicle. The excised panicles of each variety were positioned in the choice arena such that the entire panicle was available to the bugs through one of the four holes and sealed with a foam plug. Therefore, each choice arena for the first group contained four panicles, while those of the second group contained three panicles with the remaining hole plugged with a foam plug. The panicles were placed equidistant from one another. Five *O. pugnax* adults were released in the center of each arena. Each arena was treated as a single replicate, and for each group, 12 replicates were performed. In each replicate, the number of *O. pugnax* present on each rice variety was recorded at 2, 4, 6, and 20 hpr. The preference of *O. pugnax* for different rice varieties was determined by calculating the mean number of insects found on each variety at each time point.

### 4.4. Varietal Performance Study

This study examined the survival, development, and growth of *O. pugnax* on the excised panicles of eight rice varieties in clear plastic boxes (17 × 8 × 4 cm). The panicles used were taken from the rice varieties grown in the greenhouse. The excised panicles were brought to the lab on ice after inserting the cut-end portion of each panicle in separate floral tubes with water, which reduced the desiccation of the panicle. For each variety, a separate plastic box was used. Panicles at the R6/R7 stage were placed in the boxes as described in insect rearing.

One week before the initiation of the experiment, egg masses (<24 h) were collected from the laboratory colony. A single egg mass was placed in an individual cell of a rearing tray that contained a moist cotton ball and was closed with a perforated tray lid. Eggs hatched in 4 days, and after an additional 2 days, the first instars molted into second instars, which were used for the study. Second instars were used because first instars do not feed, tend to aggregate, and suffer from high mortality during handling. Six second instars were carefully transferred with a fine paint brush to a plastic box containing a panicle. Each plastic box containing six second instars was considered a single replicate, and six replicates were performed for each variety. The plastic boxes were placed randomly in an environmentally controlled room kept at 27 ± 1 °C, 14:10 h (L:D) photoperiod. Food and germination paper were changed every 3 days. To minimize genetic effects on the *O. pugnax* performance, the nymphs that developed from 3 egg masses were equally divided among all eight varieties of a single replicate. The age of *O. pugnax* nymphs was synchronized by using second instars that had molted within 6 h of each other.

Beginning ten days after the release of nymphs, the insects were checked daily at the same time in the morning until all surviving nymphs emerged as adults. The number of days taken by each instar to develop into an adult and the number of nymphs that survived to the adult stage were recorded. Newly emerged adults were removed from the plastic boxes and chilled for 30 min at 4 °C. Males were separated from females and their individual fresh weights were measured using a precision balance (Mettler–Toledo, XS105 Dual Range, Columbus, OH, USA) with a readability of 0.1 mg. For each replicate of each variety, the average nymph development time and percent survival (number of nymphs that emerged as adults divided by the total number of nymphs placed multiplied by 100) and the average adult weights of both sexes were calculated.

### 4.5. Silicon Study

This study was conducted to examine the effects of a silicon soil amendment on the performance of *O. pugnax*. The medium-grain variety Jupiter was used in this study. Rice was planted and grown in a greenhouse as described in Section 4.2, except that silicon either in the form of slag (4% Si) or wollastonite (24% Si) was administered at a rate of 1120 kg of Si/ha into the top 4–5 cm of soil mix in each pot before planting. Control plants did not receive silicon. Thus, the experiment consisted of three treatments: (1) control (no silicon); (2) slag; and (3) wollastonite. Rice planting was performed for 2 months at weekly intervals. As the heading date approached, the rice plants were observed daily, and those panicles emerging from the boot were tagged and the heading date recorded.

The procedure and environmental conditions used to conduct this experiment were identical to those described in Section 4.4 except that nine second instars (<12 h old) were placed in each plastic box and each plastic box contained a single rice panicle at the R6 or R7 stage taken from one of the three treatments. The nine instars in one plastic box were treated as a single replicate. The experiment consisted of three replicates for each treatment. Every 3 days, nymphs were provided with fresh panicles taken from the same treatment until all surviving nymphs developed into adults. For each replicate of each treatment, the percent survival, nymph development time, and adult fresh weights of both sexes were measured as described in Section 4.4.

### 4.6. Extraction of Dorsal Abdominal Glands and Metathoracic Glands

The DAG extracts of all instars were obtained using the method developed by Borges and Aldrich [23]. Briefly, fresh exuviae of each instar (within 24 h of molting to the next instar) were collected and immersed in dichloromethane for 15 min at room temperature. Following this, the extracts were transferred with a pipette to 2 mL clean glass vials. For each nymphal instar (1^st^ to 5^th^), separate extractions were performed. For each DAG extraction of first, second, third, fourth, and fifth instars, 50, 25, 10, 7, and 3 exuviae, respectively, were used. For each instar, 3 replicates were prepared. All the samples were stored at −20 °C until further use.

For the MTG extractions, 7-day-old unmated adults were used. Separate extractions were performed for males and females, and for each sex, seven samples were prepared. To extract the contents of the MTGs, adults were first anesthetized with CO_2_ for 30 s and then killed by placing them in a freezer at −20 °C. Then, each adult was dissected under a stereomicroscope in a Petri dish with wax at the bottom. Before pinning the adult, its wings and legs were removed with scissors. Then, the outer edges of the abdomen were cut and the abdominal tergites were removed. The orange-colored MTGs were exposed after the body cavity contents were removed. The contents of the MTGs were drawn out using flame-drawn microcapillary tubes and eluted by breaking the tip of the tube into hexane in a glass vial. Following this, the extract was transferred with a pipette to a clean 2 mL vial and stored at –20 °C until analysis.

The volume of each extract (DAG and MTG) was reduced to 50 µL under gentle nitrogen flow. Samples were analyzed using gas chromatography–mass spectrometry (GC–MS) with a Thermo Scientific Trace 1310 GC fitted with a TB-5 column (30 m × 0.25 mm ID × 0.25 µm film thickness) coupled with a Thermo Scientific ISQ 7000 single quadrupole mass selective detector (electron impact ionization at 70 eV). An aliquot of 1 µL of each extract was injected by an auto-sampler into the GC in spitless mode. Helium was used as the carrier gas at a constant flow rate of 1 mL/min. The initial oven temperature was set at 35 °C for 3 min, and was then increased at 7 °C/min to 280 °C. The injector and transfer line temperatures were 230 °C and 300 °C, respectively. The ion source temperature was 310 °C. The mass spectrometer was operated in scan mode (*m/z* 50–500).

The compounds in the DAGs and MTGs were tentatively identified by comparing the mass spectra with reference mass spectra in the NIST library. Some of the identified compounds from the DAGs and MTGs were further confirmed by comparing the retention times and retention indices of those compounds with a mixture of n-alkanes (C7–C40) purchased from Sigma-Aldrich, St. Louis, MI, USA. For each compound, the retention time was recorded and the retention index was calculated following the methods of Van Den Dool and Kratz [90]. To calculate the retention indices of each compound, a C7–C40 standard was injected into the GC–MS with the temperature programmed as follows: 35 °C for 3 min, ramped at 7 °C/min to 280 °C, and then increased at 20 °C/min to 320 °C and held for 2 min.

### 4.7. Data Analysis

All data were analyzed using R software [91]. A repeated-measures ANOVA using the “*lmer*” function was performed to determine the varietal preference of *O. pugnax*. In each test, the variety and time points were within subject factors and the arena was a random effect. The Sidak test was used for mean comparisons. For both the varietal performance study and the silicon study, each replicate was treated as a block following a randomized complete block design. The initial analysis showed that nymph development time did not differ between males and females; hence, the data were pooled for the final analysis. The data on nymph development time, percent survival to adult stage, and adult weights of both sexes were analyzed separately using one-way ANOVA with treatment included as a fixed effect and replicate as the block. When the treatment effect was significant, mean comparisons were performed using Tukey’s HSD test. Residual normality and the homogeneity of variances were verified by the Shapiro–Wilk test and Levene’s test, respectively.

## 5. Conclusions

In summary, this study showed some differences in the preference of adult *O. pugnax* for rice varieties, with the variety Cheniere preferred over Kaybonnet. Further investigations elucidating the mechanisms that mediate the preference of *O. pugnax* are required. Such knowledge may be useful in breeding resistant or less-susceptible varieties. Additionally, this is the first study to demonstrate the negative impact of silicon amendment on *O. pugnax* survival and development. Further studies are required to examine if poor nymph performance translates to less insect feeding and to lower yield losses and incidence of peck under field conditions, which will aid in providing recommendations for silicon application in commercial rice fields to control *O. pugnax*. Further in this study, the chemical compositions of the metathoracic glands and dorsal abdominal glands of *O. pugnax* were characterized for the first time. This information can be used to develop chemical compounds for the management of this species through sustainable and eco-friendly practices.

## Figures and Tables

**Table 1:** Mean (±SE) nymph development time (days), survival to adult stage (%), and adult fresh weights (mg) of 1 d-old male and female *Oebalus pugnax* reared on eight rice (*Oryza sativa*) varieties in the laboratory at 27 ± 1 °C and 14:10 h L:D photoperiod.

Rice Variety	Development Time	Survival	Male Weight	Female Weight
Cheniere	16.25 ± 0.35	88.88 ± 5.55	33.17 ± 0.79	44.33 ± 2.04
CL151	16.27 ± 0.18	91.67 ± 5.69	34.17 ± 0.60	43.60 ± 1.03
Frontiere	16.38 ± 0.26	91.67 ± 5.69	32.33 ± 0.56	43.33 ± 1.23
Jazzman	16.28 ± 0.32	77.77 ± 10.25	31.80 ± 1.07	44.67 ± 1.74
Jupiter	16.03 ± 0.22	83.33 ± 11.39	34.50 ± 1.75	44.33 ± 2.25
Kaybonnet	16.63 ± 0.21	75.02 ± 8.33	32.83 ± 1.05	43.67 ± 1.58
LaGrue	16.42 ± 0.40	88.88 ± 5.55	33.33 ± 0.76	44.00 ± 1.00
Mermentau	16.47 ± 0.40	88.87 ± 3.52	30.83 ± 1.25	39.83 ± 1.58

Means in each column did not differ significantly (*p* > 0.05).

**Table 2:** Relative abundance (%) (mean ± SE) of different compounds present in the exuviae of the dorsal abdominal gland extracts of first through fifth instars of the rice stink bug, *Oebalus pugnax*.

Group	Compound	1st Instar	2nd Instar	3rd Instar	4th Instar	5th Instar
Alkane	Decane *	ND	ND	0.43 ± 0.22	ND	ND
	Undecane *	ND	ND	ND	0.31 ± 0.07	0.19 ± 0.03
	Dodecane *	0.28 ± 0.05	0.28 ± 0.05	0.48 ± 0.19	0.73 ± 0.36	0.71 ± 0.30
	Tridecane *	26.17 ± 2.07	30.45 ± 1.95	30.83 ± 5.68	26.74 ± 5.41	22.41 ± 5.51
	Tetradecane *	ND	ND	ND	ND	0.09 ± 0.04
	Pentadecane *	ND	ND	ND	ND	0.13 ± 0.06
						
Alkene	1-Tridecene ^†^	ND	0.11 ± 0.01	0.10 ± 0.06	0.30 ± 0.15	0.42 ± 0.18
						
Aldehyde	(*E*)-2-Hexenal ^†^	ND	ND	0.24 ± 0.13	0.26 ± 0.12	0.50 ± 0.30
	Cinnamaldehyde ^†^	0.20 ± 0.08	ND	ND	ND	ND
	2-Undecenal ^†^	26.67 ± 1.57	0.65 ± 0.01	0.06 ± 0.03	0.08 ± 0.03	ND
	Tridecanal ^†^	ND	0.09 ± 0.01	0.11 ± 0.07	0.31 ± 0.18	ND
	Tetradecanal ^†^	8.43 ± 1.48	24.28 ± 1.93	20.99 ± 6.04	17.86 ± 4.72	22.85 ± 5.35
						
Oxo-alkenal	(*E*)-4-Oxo-2-hexenal ^†^	35.57 ± 0.66	43.30 ± 4.08	46.74 ± 5.87	53.37 ± 10.17	52.69 ± 11.01
						
Terpenoids	Linalool ^†^	2.33 ± 0.90	0.77 ± 0.57	ND	ND	ND
	α-terpineol ^†^	0.42 ± 0.11	ND	ND	ND	ND

Identifications were tentatively made by comparing the mass spectra of each compound with the NIST mass spectra library. * Compounds were also identified by comparing retention times with a C7–C40 alkane standard mixture. † Compounds were also identified by retention indices prepared using a C7–C40 alkane standard mixture. For each instar, the relative abundance of each compound was the mean of three samples. ND—not detected.

**Table 3:** Relative abundance (%) (mean ± SE) of different compounds found in the metathoracic gland extracts of adult rice stink bugs, *Oebalus pugnax*.

Group	Compound	Retention Time (min)	Retention Index	Male	Female
Alkane	Octane *	6.7	801	0.04 ± 0.02	0.08 ± 0.05
	Undecane *	13.9	1099	1.41 ± 0.39	1.16 ± 0.57
	Dodecane *	16.1	1201	4.80 ± 0.38	5.16 ± 1.86
	Tridecane *	18.2	1304	37.46 ± 3.53	27.57 ± 4.87
	Tetradecane *	20.0	1399	0.72 ± 0.17	0.76 ± 0.17
	Pentadecane *	21.7	1499	1.33 ± 0.22	1.12 ± 0.37
	Hexadecane *	23.5	1601	0.02 ± 0.01	0.03 ± 0.01
	Heptadecane *	25.0	1699	0.02 ± 0.01	0.03 ± 0.01
	Octadecane *	26.5	1798	0.01 ± 0.00	0.02 ± 0.01
					
Alkene	1-Tridecene ^†^	17.9	1292	1.32 ± 0.14	1.43 ± 0.32
	1-Tetradecene ^†^	18.7	1330	0.03 ± 0.01	0.05 ± 0.02
	1-Pentadecene ^†^	21.6	1492	0.07 ± 0.03	0.04 ± 0.01
					
Alcohol	3-Hexanol ^†^	6.6	795	0.04 ± 0.01	0.07 ± 0.02
	(*E*)-2-Octenol ^†^	13.2	1070	0.70 ± 0.18	2.51 ± 0.87
	(*E*)-2-Decenol ^†^	17.5	1271	0.29 ± 0.07	2.29 ± 1.89
					
Aldehyde	(*E*)-2-Hexenal ^†^	8.1	853	4.62 ± 0.89	7.89 ± 1.37
	(*Z*)-2-Heptenal ^†^	10.6	959	0.02 ± 0.01	0.04 ± 0.01
	(*E*)-2-Octenal ^†^	13.1	1062	13.54 ± 1.15	9.46 ± 2.22
	(*E, E*)- 2,4-Octadienal ^†^	14.2	1113	ND	0.02 ± 0.02
	(*E*)-2-Nonenal ^†^	15.3	1164	0.04 ± 0.01	0.05 ± 0.04
	(*Z*)-4-Decenal ^†^	16.0	1199	ND	0.60 ± 0.58
	(*Z*)-2-Decenal ^†^	17.4	1266	5.33 ± 0.79	3.84 ± 1.71
	Tetradecanal ^†^	23.7	1615	0.04 ± 0.02	0.06 ± 0.02
					
Oxo-alkenal	(*E*)-4-Oxo-2-hexenal ^†^	10.9	970	12.07 ± 1.22	12.26 ± 2.57
					
Ketone	3-Hexanone ^†^	6.4	784	0.02 ± 0.00	0.02 ± 0.01
	2-Hexanone ^†^	6.5	789	0.16 ± 0.04	0.23 ± 0.06
	6-Tridecanone	21.4		0.05 ± 0.01	0.05 ± 0.02
	4-Tridecanone	21.3		0.11 ± 0.02	0.10 ± 0.04
					
Esters	(*Z*)-2-Hexenyl acetate ^†^	12.0	1014	0.04 ± 0.02	1.90 ± 0.89
	(*E*)-2-Heptenyl acetate ^†^	14.3	1116	ND	0.01 ± 0.01
	1-Octenyl-3-acetate	16.4		8.73 ± 1.20	14.60 ± 2.66
	(*E*)-2-Decenyl acetate ^†^	20.2	1410	6.97 ± 0.43	6.53 ± 0.78
	Isopropyl palmitate ^†^	29.7	2014	0.01 ± 0.00	0.01 ± 0.01

Identifications were tentatively made by comparing the mass spectra of each compound with the NIST mass spectra library. * Compounds were also identified by comparing retention times with a C7–C40 alkane standard mixture. † Compounds were also identified by retention indices prepared using a C7–C40 alkane standard mixture. Sample size = 7 for both sexes. ND—not detected.

## Data Availability

All the data are available from the corresponding author upon reasonable request.
